# Supplementary material for: Structure and Dynamics of the Unassembled Nucleoprotein of Rabies Virus in Complex with Its Phosphoprotein Chaperone Module
Source: Viruses. 2022 Dec 16;14(12):2813. doi: 10.3390/v14122813 (PMC9786881; doi:10.3390/v14122813)
Supplement: Supplementary file 1 [file viruses-14-02813-s001.zip › viruses-2038678-supplementary.pdf]

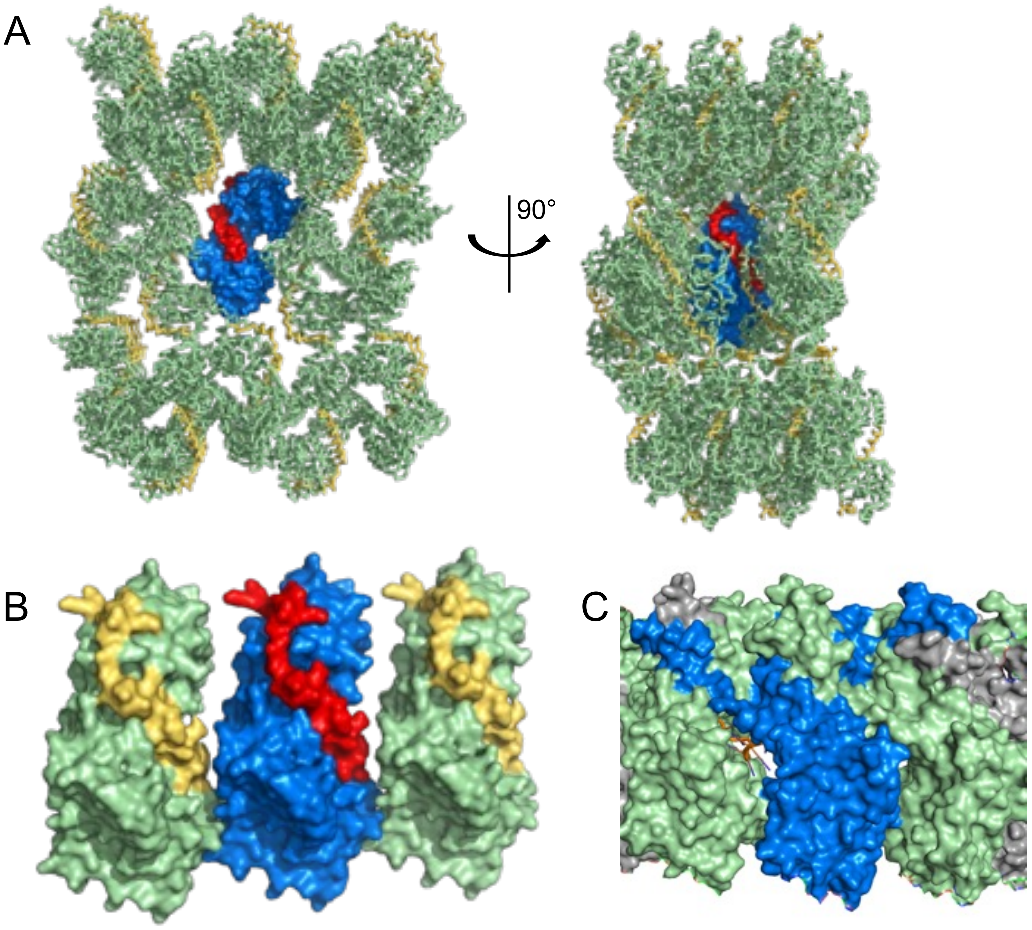

Figure S1. Absence of N polymerization in the crystal of the $N_{\Delta 23}^{0}$-P_{68} complex. (A) Crystal packing shown in two orthogonal orientations. One heterodimer is shown in surface representation and colored differently than the others, with $N_{\Delta 23}$ shown in blue and P_{68} in red. The heterodimer in front of the one colored in blue/red has been removed for clarity. (B) Close-up on a linear array of three adjacent heterodimers, showing the limited contacts between N_{NTD}, but the absence of contact between N_{CTD}. (C) Close-up of adjacent protomers in the circular N-RNA complex, showing a different orientation of the protomers with limited contacts between N_{NTD}, but more extensive contacts between N_{CTD}, involving the $N_{NT\text{-}ARM}$ of the N_{i-1} subunit and the $N_{CT\text{-}ARM}$ of the N_{i+1} subunit).

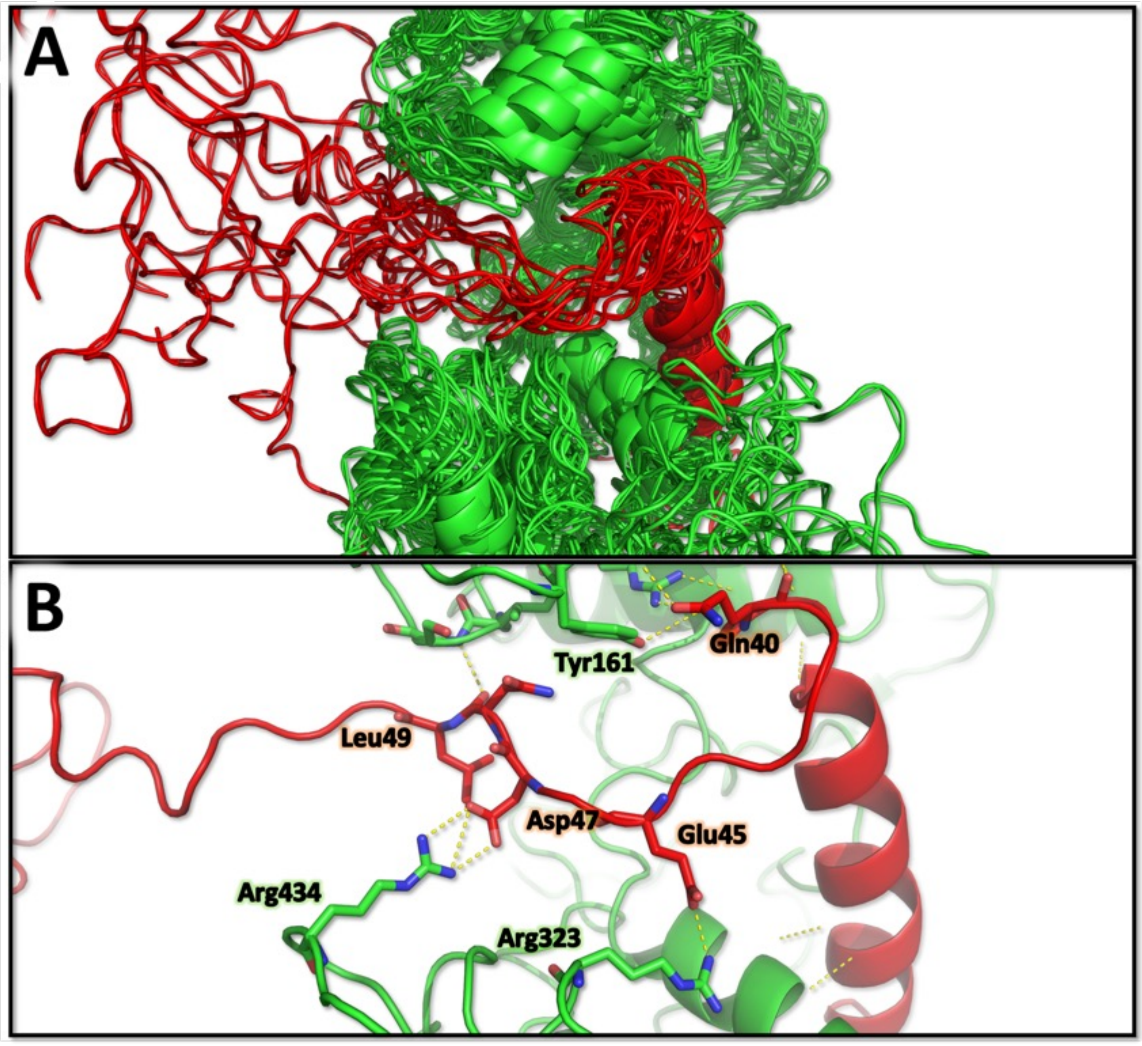

Figure S2. Computational models extracted from MD simulations showing transient interactions between the C-terminal flexible part of P_{68} and $N_{\Delta 23}$. (A) Superposition of different snapshots taken from the MD simulations. Cartoon representation of $N_{\Delta 23}$ shown in green and P_{68} in red. showing the region of P68 inserted in the RNA binding groove (B) Close-up of an individual transient conformers (one snapshot taken from the MD simulation) showing the possible polar contacts between P_{68} and basic residues in the RNA-binding groove of N.

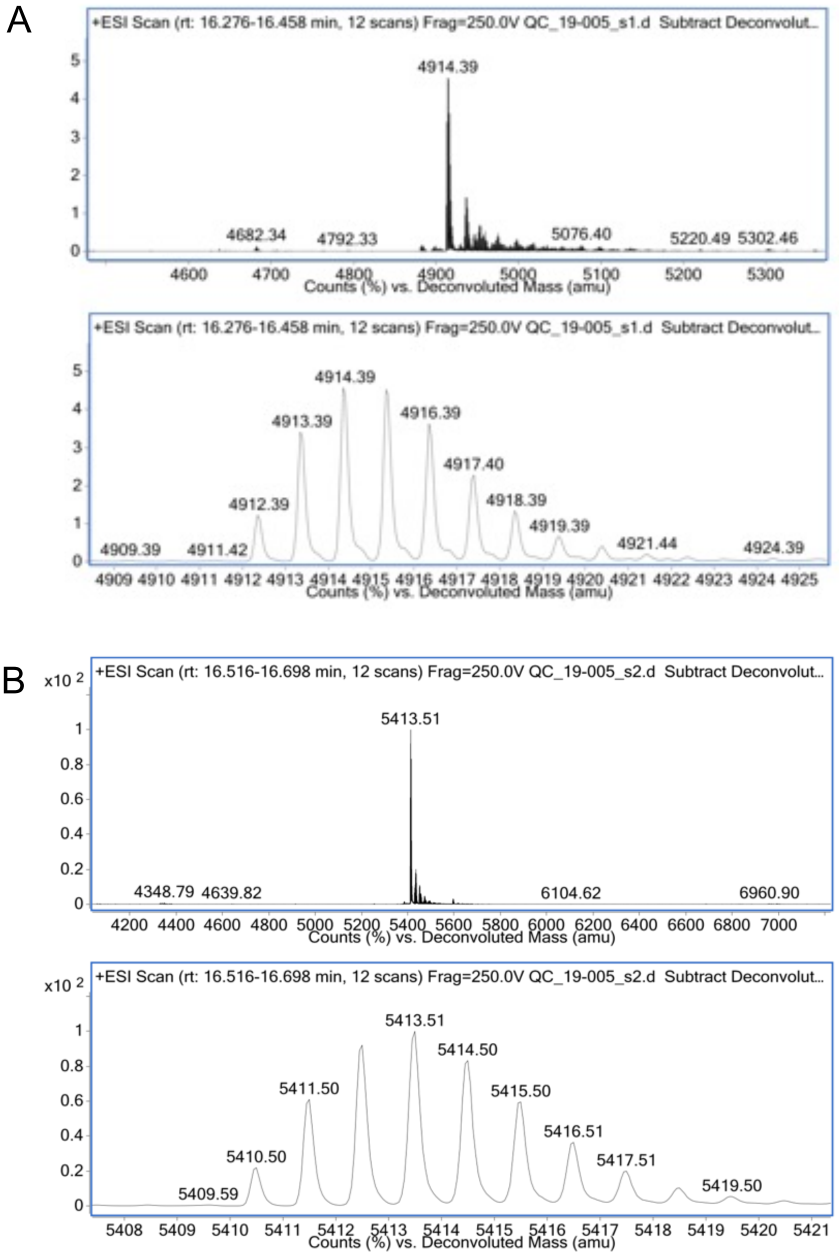

Figure S3. Assessment of the labeling of P_{42} by FAM by mass spectrometry. (A) Electro-spray ionization (ESI) mass spectrometry of unlabeled P_{42}. The upper panel shows and the lower panel shows one spectrum showing the different ions. The theoretical monoisotopic molecular mass calculated from the sequence is 4912.38 Da and the experimental value is 4912.39 ± 0.1 Da (B) Electro-spray ionization (ESI) mass spectrometry of FAM-labeled P_{42}. The upper panel shows and the lower panel shows one spectrum showing the different ions. The theoretical monoisotopic molecular mass calculated from the sequence is 4912.38 + 498.11 = 5410.49 Da and the experimental value is 4910.50 ± 0.1 Da .

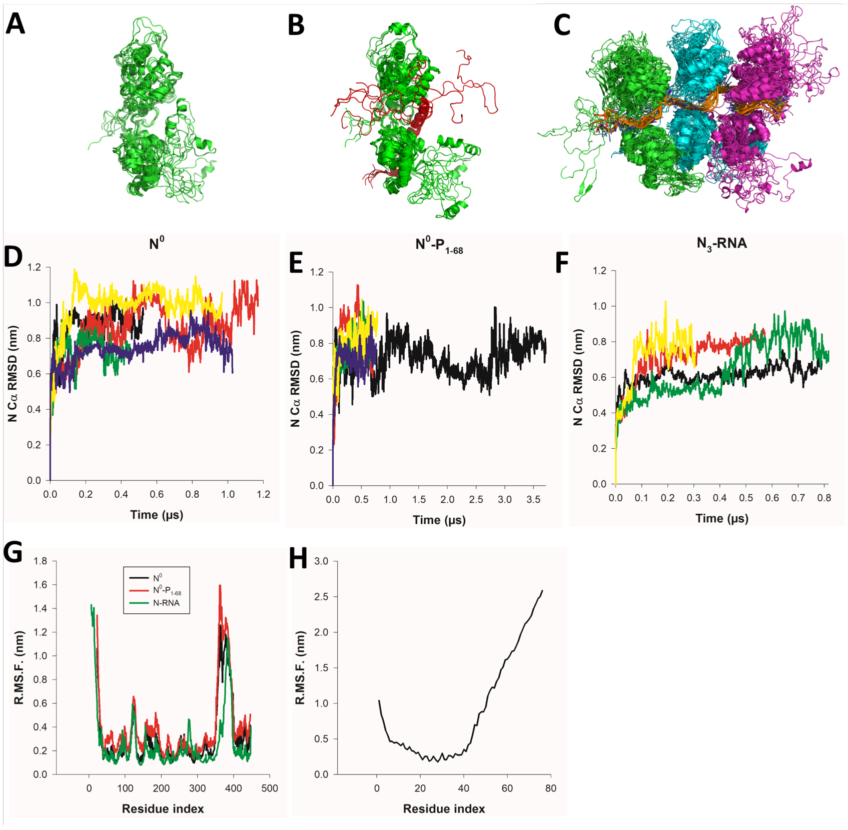

Figure S4. Movements in $N_{\Delta 23}$ alone, in $N_{\Delta 23}$ in complex with P_{68} and in assembled full-length N in complex with RNA. (A) Superposition of snapshots taken from the MD simulations shown in panel A. $N_{\Delta 23}$ is colored in green. (B) Superposition of snapshots taken from the MD simulations shown in panel B. $N_{\Delta 23}$ is colored in green and P_{68} in red. (C) Superposition of snapshots taken from the MD simulations shown in panel C. The three subunits of N are shown in different colors. (D) Plots of the root mean square deviation (RMSD) as a function of the simulation time from independent trajectories, with respect to the starting coordinate of $N_{\Delta 23}$ alone extracted from the $N_{\Delta 23}$-P_{68} crystal structure (8b8v). (E) Plots of the root mean square deviation (RMSD) as a function of the simulation time from independent trajectories, with respect to the starting coordinate of $N_{\Delta 23}$-P_{68} crystal structure (8b8v). **(**F) Plot of the root mean square deviation (RMSD) as a function of the simulation time, with respect to the starting coordinate of a N_3-RNA system extracted from the re-refined crystal structure (circular N-RNA complex – 2GTT.pdb). (G) Plot of the root mean square fluctuations (RMSF) in N calculated over each MD simulation. (H) Plot of the root mean square fluctuations (RMSF) in P_{68} calculated over the MD simulations of the $N_{\Delta 23}$-P_{68} complex shown in panel B.

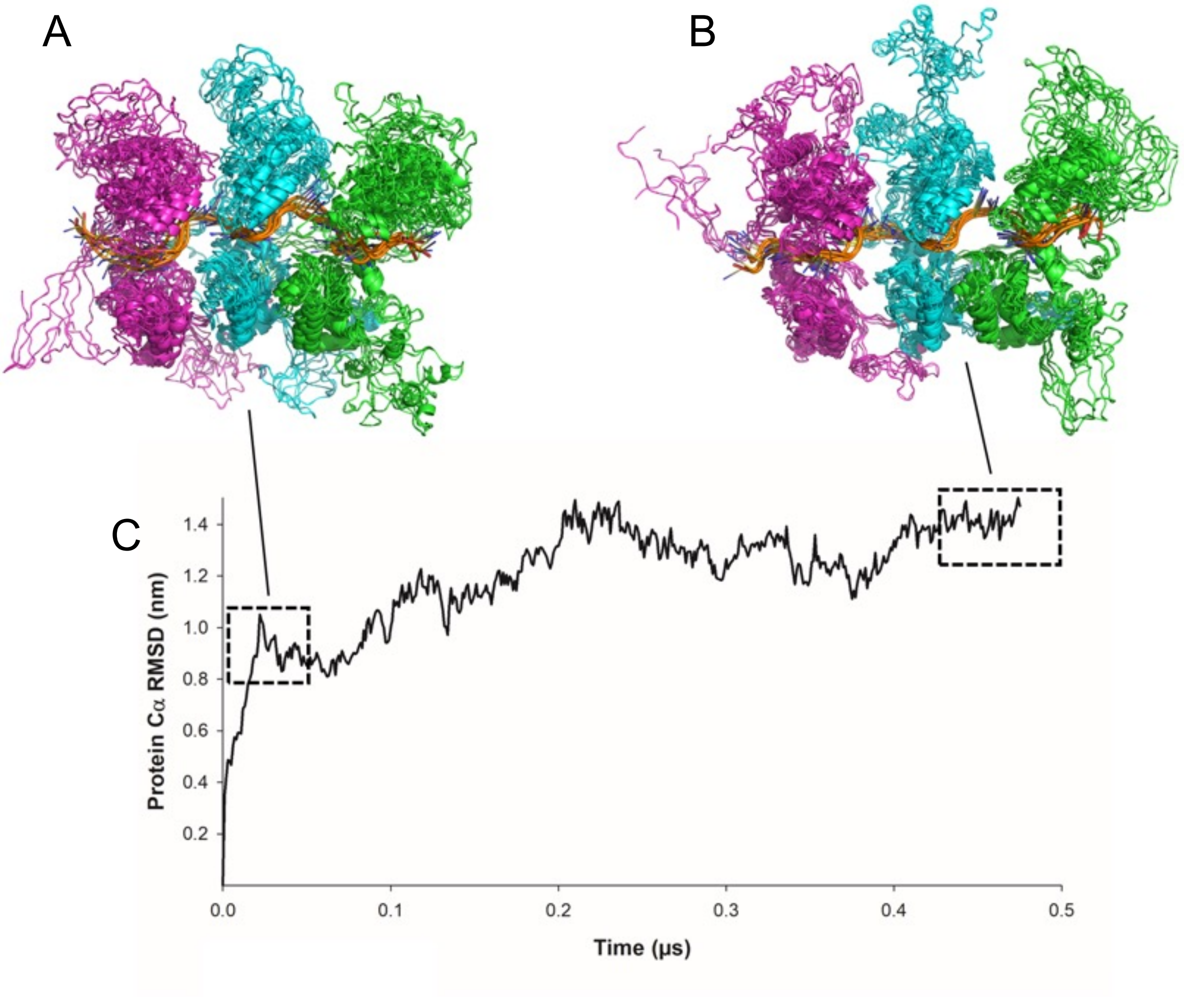

Figure S5. Unfolding of the nucleoprotein in molecular dynamics simulations revealed defects in the structural model. (A) Plot of the root mean square deviation (RMSD) as a function of the simulation time, with respect to the starting coordinate of a N_3-RNA system extracted from the crystal structure (circular N-RNA complex – 2gtt). (B) Superposition of snapshots taken at the beginning of the MD simulation. The three N subunits and the RNA molecules are shown in cartoon representation in different colors. (C) Superposition of snapshots taken at the end of the MD simulation. The three N subunits and the RNA molecules are shown in cartoon representation in different colors.

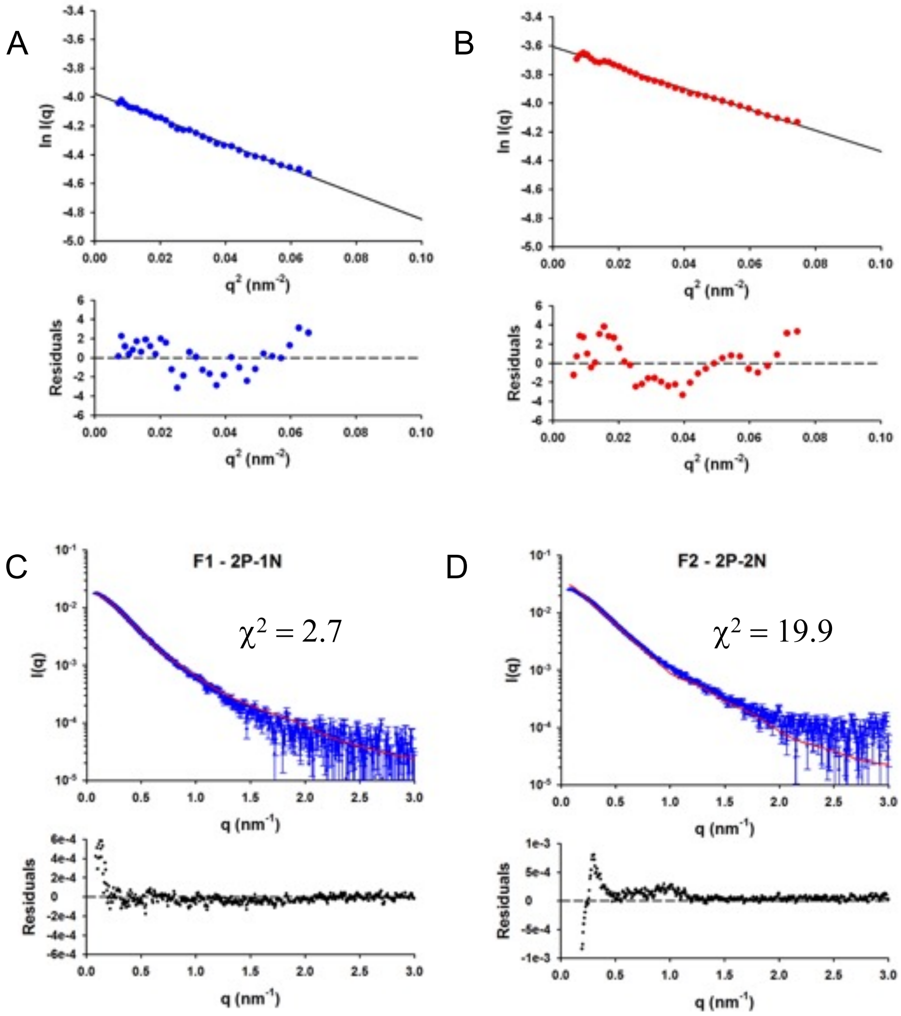

Figure S6. SEC-SAXS of $N_{\Delta 23}{}^{0}$-P_{FL} complex. (A) Guinier plots for F1. The red line in the upper panel shows the linear fit. The lower panel shows the plot of the residuals. (B) Guinier plots for F2. The red line in the upper panel shows the linear fit. The lower panel shows the plot of the residuals. (C) Conformational ensemble modeling by the ensemble optimization method (EOM). The upper panel show the experimental F1scattering profile (blue circles) The red line shows back-calculated scattering curve for a selected ensemble of 4 2P-1N conformers shown in panel E (χ^2 = 2.7). The lower panel shows the plot of the residuals (black symbols). (D) Conformational ensemble modeling by the ensemble optimization method (EOM). The upper panel show the experimental F2 scattering profile (blue circles) The red line shows back-calculated scattering curve for a selected ensemble of 4 2P-2N conformers shown in panel E (χ^2 = 19.9). The lower panel shows the plot of the residuals (black symbols).

Table S1. Polar contacts between $N_{\Delta 23}$ and P_{68} in the crystal. Hydrogen bonds and salt bridges were identified with the server PISA.

Hydrogen Bonds			
##	P1-68	Dist. [Å]	N
1	B:VAL 6[N]	2.90	A:PHE 261[O]
2	B:ARG 12[NE]	3.46	A:GLU 403[OE2]
3	B:ARG 12[NH2]	3.38	A:GLU 403[OE2]
4	B:ARG 12[NH2]	3.40	A:GLU 403[OE1]
5	B:THR 24[OG1]	2.72	A:THR 279[OG1]
6	B:ASN 29[ND2]	3.14	A:THR 243[O]
7	B:ASN 31[ND2]	2.91	A:TYR 233[OH]
8	B:ILE 4[O]	2.94	A:LYS 263[N]
9	B:GLY 14[O]	3.24	A:ARG 254[NH2]
10	B:LEU 15[O]	2.40	A:ARG 254[NH2]
11	B:GLU 22[OE1]	3.35	A:ARG 254[N]
12	B:GLU 22[OE2]	2.81	A:ALA 253[N]
13	B:ASN 29[OD1]	2.95	A:THR 243[OG1]
14	B:ASN 29[OD1]	3.55	A:GLY 244[N]
15	B:ASN 35[OD1]	3.01	A:ARG 225[NH2]
16	B:HIS 38[O]	3.49	A:ARG 149[NH1]
Salt bridges			
##	P1-68	Dist. [Å]	N
1	B:ARG 12[NE]	3.46	A:GLU 403[OE2]
2	B:ARG 12[NH2]	3.38	A:GLU 403[OE2]
3	B:ARG 12[NH2]	3.40	A:GLU 403[OE1]
